# The impact of particle radiotherapy on the functioning of cardiac implantable electronic devices: a systematic review of in vitro and in vivo studies according to PICO criteria

**DOI:** 10.1007/s11547-022-01520-6

**Published:** 2022-07-24

**Authors:** Amelia Barcellini, Veronica Dusi, Alfredo Mirandola, Sara Ronchi, Giulia Riva, Francesca Dal Mas, Maurizio Massaro, Viviana Vitolo, Mario Ciocca, Roberto Rordorf, Ester Orlandi

**Affiliations:** 1 Radiation Oncology, Clinical Department, National Center for Oncological Hadrontherapy (CNAO), Pavia, Italy; 2 Coronary Care Unit and Laboratory of Clinical and Experimental Cardiology, Fondazione IRCCS Policlinico San Matteo, Pavia, Italy; 3 Division of Cardiology, Department of Medical Sciences, AOU Città Della Salute e Della Scienza, University of Turin, Turin, Italy; 4 Department of Management, Ca’ Foscari University of Venice, Venice, Italy

**Keywords:** Cardiac implantable electronic device, Carbon ion radiotherapy, Malfunctions, Pacemaker, Implantable cardioverter defibrillator, Proton beam radiotherapy

## Abstract

The number of oncological patients who may benefit from proton beam radiotherapy (PBT) or carbon ion radiotherapy (CIRT), overall referred to as particle radiotherapy (RT), is expected to strongly increase in the next future, as well as the number of cardiological patients requiring cardiac implantable electronic devices (CIEDs). The management of patients with a CIED requiring particle RT deserves peculiar attention compared to those undergoing conventional photon beam RT, mostly due to the potential generation of secondary neutrons by particle beams interactions. Current consensus documents recommend managing these patients as being at intermediate/high risk of RT-induced device malfunctioning regardless of the dose on the CIED and the beam delivery method used, despite the last one significantly affects secondary neutrons generation (very limited neutrons production with active scanning as opposed to the passive scattering technique). The key issues for the current review were expressed in four questions according to the Population, Intervention, Control, Outcome criteria. Three in vitro and five in vivo studies were included. Based on the available data, PBT and CIRT with active scanning have a limited potential to interfere with CIED that has only emerged from in vitro study so far, while a significant potential for neutron-related, not severe, CIED malfunctions (resets) was consistently reported in both clinical and in vitro studies with passive scattering.

**Supplementary Information:**

The online version contains supplementary material available at 10.1007/s11547-022-01520-6.

## Introduction

As a result of aging and comorbidities, the incidence of cancer and the use of cardiac implantable electronic devices (CIEDs) have increased concomitantly [1, 2]. A large Danish population-based cohort study including patients undergoing conventional photon beam RT reported that the annual rate of RT courses in pacemaker (PM)/implantable cardioverter–defibrillator (ICD) patients in Western Denmark was 4.33 per 100 000 person-years in 2012, representing an increment of 199% since 2003 [3].

As previously reported [4, 5], RT can induce CIEDs malfunctions as a result of several factors, including (1) stochastic effects associated with secondary neutrons interactions; (2) transient noise oversensing due to the RT dose rate used, with dose rates < 0.01 Gy/min considered at low risk; (3) the total cumulative dose delivered to the device's generator, with doses below 2 Gray (Gy) considered at low risk. Secondary neutrons can be produced, according to the RT type, energy and delivery method, by the nuclear interactions with the material in the beam path during treatment. These factors can lead to three types of CIED malfunctions: (1) *Transient malfunctions,* occurring only during RT exposure because of electromagnetic interferences; (2) *Reverting to back-up* setting (reset), a condition that can be either recovered after CIED programming or spontaneously resolve; (3) *Permanent* malfunctions, demanding CIED replacement. From a functional point of view, CIED malfunctions can be classified into hard errors due to damage to the hardware and most often related to direct irradiation and soft errors, due to software (random access memory) alterations and usually associated with scattered radiation or electromagnetic interference, being the reset a typical example of the latter group [6, 7]. Notably, except for a single case report in which a shock coil failure secondary to external irradiation was reported [8], there is no solid evidence in the literature suggesting a significant potential for a negative effect of RT on the electrodes in CIED systems. On the contrary, dedicated reports confirming the safety of RT on ICD leads despite high RT dosages (> 50 Gy) exist [9]. Currently, there is no safe threshold dose for the electrodes, the leads and the coils that are generally considered to be insensitive to radiation.

Proton beam therapy (PBT) and carbon ion RT (CIRT) have recently emerged as encouraging RT methods to limit radiation toxicity associated with conventional RT with photons while maintaining high rates of tumor local control for several malignancies [10–13]. Heavy ions such as protons and carbon ions have physical and biological advantages over photons: the so-called inverse dose deposition of particle beams allows the deposition of the majority of the energy at the end of their path in tissue, with limited dose in the entrance channel and beyond the target [11]. Moreover, their higher relative biological effectiveness (RBE) compared to photons allows delivering low physical dose to obtain the required therapeutic dose to the target. Notably, despite the limited available studies, in current consensus documents [13–16] patients with an implanted CIED and receiving PBT were considered at intermediate/high risk of RT-induced device malfunctioning, independently on the beam delivery method, on the estimated dose on the generator and on the generator and the tumor site. This assumption was based on the expected larger amount of secondary neutrons production by PBT as compared to conventional RT with energies below 10 MeV. Except for the recent European consensus document [16] that assigns to CIRT a low potential for RT-induced device mulfunctioning without providing any reference, no specific recommendations were provided for patients receiving CIRT, although there was a general warning to consider at high-risk patients receiving RT with a high potential for secondary neutrons production. The German guidelines express concerns about the delivery of PBT in patients with CIEDs without suggesting practical management strategies [17]. Finally, the recently released Polish expert opinion and consensus paper about cancer patients with CIEDs undergoing RT does not even address the issue of PBT and CIRT [18]. Of note, the amount of secondary neutron production induced by both PBT and CIRT is critically influenced by the beam delivery method, namely active scanning modality (also referred to as pencil beam scanning or spot scanning, in which a beam of particles is magnetically scanned across the field) rather than passive scattering (in which the beam is spread out laterally as a uniform beam through single or double scattering). The former is associated with very limited secondary neutron production [19, 20]. Nonetheless, 2017 American Guidelines recommend (Class I, level of evidence B not randomized) to prefer non-neutron-producing treatment over neutron-producing treatment in patients with a CIED to minimize the risk of device reset, therefore potentially depriving CIED patients of a life-saving treatment from an oncological point of view due to the presence of a CIED. On the contrary, patients receiving conventional RT are generally considered at high risk only in case of high dosages to the generator, of high energy photons, of PM dependency of the patient combined to the usage of high-energy photons, or of the presence of an ICD, particularly in case of a positive history for previous appropriate interventions. A recent paper shares the same approach [21]. Notably, for patients considered at high risk, intensive intra treatment monitoring and consideration for CIED relocation are recommended [13–15, 17, 18, 22].

Starting from these premises, the present work aims to review the literature data about CIEDs malfunction associated with PBT and CIRT and to provide an overview of the studies investigating the management of patients with CIEDs undergoing PBT or CIRT.

## Material and methods

### Search strategy

**Fig. 1:**
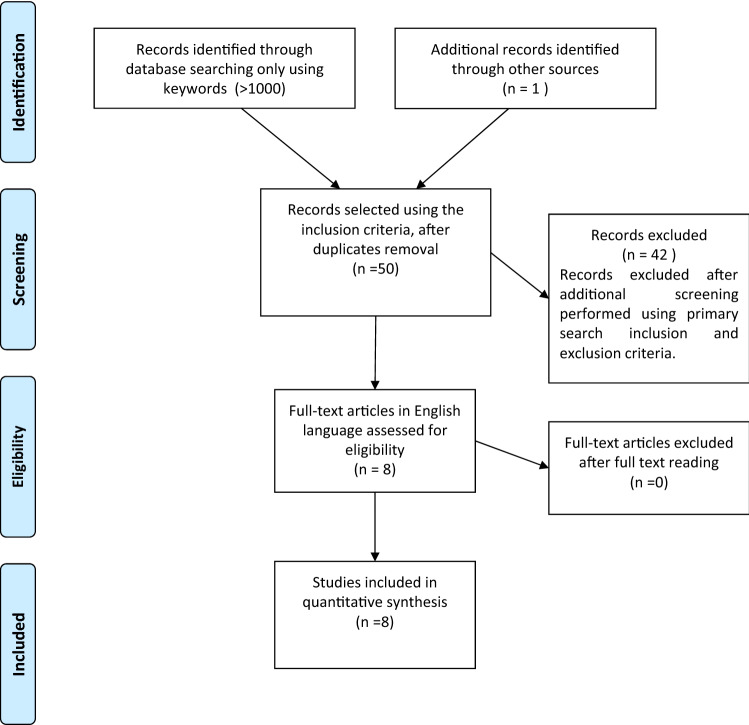
PRISMA flow diagram of study selection process

The key issue was expressed in four questions according to the Population, Intervention, Control, Outcome (PICO) design approach [23, 24]. These queries (Table 1) have been the matter of a literature search in the PubMed, Web of Science, and Scopus databases from 2000 to 2021 according to a combination of the following keywords: "Defibrillator," “Implantable Cardiac Device,” “Pacemaker,” “Radiotherapy,” “Hadrontherapy,” “Particle,” “Carbon Ions,” “Heavy Ions,” “Protons” including pluralization and US English/UK English spelling variations and suffixes/prefixes. We conducted a systematic search using the Preferred Reporting Items for Systematic Reviews and Meta-Analyses (PRISMA) literature selection process (Fig. 1) [25]. Two authors independently searched reference lists of recognized manuscripts to integrate the literature search.

**Table 1:** Research quests according to PICO criteria

Query	Population	Intervention	Comparison	Outcomes
#1	In vitro malfunctioning	Protons and Carbon ions	None	Intra- and inter-session occurrence of CIED malfunction (type and incidence) and potential of perturbation of the delivered beam by CIED leads
#2	In vivo malfunctioning	Protons and carbon ions	None	Intra- and inter-session occurrence of CIED malfunction (type and incidence)
#3	In vivo malfunctioning	Protons and carbon ions	None	Malfunction management
#4	In vivo malfunctioning	Protons and carbon ions	None	Relationship with the treatment planning (total dose to CIED, field-to-generator distance, dose range, distance between CIED and target volume, type of particle, type of scanning)

### Selection criteria for full-text article review

Papers were suitable for insertion in the review if the following criteria were fulfilled: (1) published as a full article in peer-reviewed journals; (2) PBT and CIRT techniques; (3) in vivo and/or in vitro study, (4) at least one of the considered outcomes (type of malfunction/management of malfunction/relationship with planning) reported; (5) articles written in the English language. Interventional, observational, prospective and retrospective studies were considered. Exclusion criteria were: (1) RT with other RT modalities than PBT or CIRT (brachytherapy, intraoperative RT, photon beam RT, electron beam RT); (2) single case reports, book chapters, books, or Conference Proceedings. A total of 8 publications met the inclusion and exclusion criteria, for a total of 3 in vitro studies and 5 in vivo studies.

### Risk of bias

For each selected study, two investigators independently assessed the risk of bias to ensure validity and overcome eventual selection, performance, detection, attrition and reporting bias, according to Cochrane Handbook for Systematic Reviews of Interventions [26–28]. Bias across studies as well as bias and risks related to the source of funding and conflict of interest of authors of the included studies was assessed. Eventual disagreements were resolved through discussion.

### Data collection

The data extraction form was validated by two researchers, and data were independently extracted by the two researchers.

### Statistical analysis

Continuous variables are presented as  mean ± standard deviation or as median (interquartile range [IQR]), while categorical variables are reported using counts and percentages. To compare  variables, the Student’s t–test or the Mann-Whitney U test for continuous variables and the Chi-square or the Fischer exact test for categorical variables are used. A p value <0.05 was considered statistically significant.

## Results

The selection of studies analyzed in the present review is shown in Fig. 1. Tables 2 and 3 show the studies characteristics; Supplemental Table 1 reports the risk of bias appraisal; Table 4 synthesizes the outcomes of the selected clinical studies.

**Table 2:** Key characteristics and description of the in vivo studies included

Authors	N of pts	Age (mean ± SD)	Thoracic tumor (%)	ICD/ PM (%)	PM dependence (%)	Year of Implant	Device brand	Intrasession monitoring	CIED check	Type of RT (%)	N of fractions	Total median dose (range) (GyE)	Active /passive scanning beams (%)
Oshiro et al. [29]	8	77.5 ± 5.2 (range 66–81)	12	0/100	62 (defined as VP ≥ 98%)	Range 1984–2004	3 MDT, 1 Guidant, 1 intermedics, 2 SJM, 1 Biotronik	Continuous ECG	Weekly	PBT	127	(33–77)	0/100
Gomez et al. [30]	42	NA	59	33/67	7	NA	NA	In-room video system and pulse monitoring	After each session	PBT	1352	74 (46.8–87.5)	24/76
Ueyama et al. [31]	7	80.1 ± 6.7 (range 74–90)	43	0/100	NA for all cases	NA	3 MDT, 2 SJM, 2 Boston	Continuous ECG	After each session	PBT	NA	NA	0/100
Seidensaal et al. [32]	31	72 (range 43–89)	13	10/90	23% dependents (heart rate < 30 bpm), 6% missing	NA	19 MDT, 4 SJM, 4 Biotronik, 3 Boston, 1 Ela Medic	In-room video, audio and motion system	After each session	68 CIRT, 29 PBT, 3 both	504	51 (10–66)	100/0
Hashimoto T et al. [33]	69	Median 81 (range 60–97)	29	7/93	NA	NA	21 MDT 10 SJM 5 Biotronik 6 Guidant 5 Vitatron 1 Boston 1pacesetter 1 Sorin 19 Unk	NA	NA	67 PBT, 32 CIRT, 1 both	NA	Range 33.3–88	0/100
Overall population	157	Range 43–97	34	14/86	Mostly NA	Mostly NA	n = 96, mostly MDT (48%) and SJM (17%)	n = 88, 83% in room monitoring17% continuous ECG	n = 88, 48% beginning and end of RT, 43% after each session, 9% weekly	79% PBT, 20% CIRT, 1% both	N = 1983 available	Range 10–88	26/74

*CIRT *carbon ion radiotherapy, *ICD* implantable cardioverter defibrillator, *MDT* Medtronic, *NA* not available, *PBT* proton beam therapy, *PM* pacemaker, *SJM* St Jude Medical (Abbott), *VP* ventricular pacing

**Table 3:** Key characteristics and description of the in vitro studies included

Study	Population			Intervention		
Authors	Type of study	Number of ICDs/leads	Intrasession monitoring	Type of RT	Dose in field	ICD/lead position
Hashimoto et al. [34]	set-up study for a patient with an ICD	4 ICD generators (Marquis DR 7274, Medtronic)	ICDs were checked after each radiation fraction	Passive scattering PBT	Total dose in-field of 107 Gy in 10 fractions	ICDs were placed in the phantom (0.3 cm laterally and 3 cm distally) outside the RT field of 10 × 10 cm2
Wootton et al. [35]	perturbation to deliver proton beam RT to a water phantom containing a high-voltage ICD lead	1 ICD lead	–	PBT	200 cGy dose with a 10X10 cm treatment field and a 10 cm wide spread-out Bragg peak (SOBP)	The ICD lead was placed at both the center and the distal edge of a clinical spread-out Bragg peak (SOBP) in a water phantom, in both a stationary position and with the lead moving in a periodic pattern to simulate cardiorespiratory movement
Bjerre et al. [36]	experimental in vitro study investigated risk of CIED errors during pencil beam proton therapy	62 explanted CIEDs from 4 manufacturers (Biotronik, Boston Scientific, Medtronic, and St. Jude Medical)	13 devices, with leads connected, were monitored live during consecutive irradiations; the remaining 49 were checked after each radiation fraction	Active scanning PBT	2 Gy in a field of 10 × 10 × 10 cm SOBP, using energies from 161.4 to 214.7 MeV	The vertical distance from the distal edge of the SOBP to the device was a constant 3 cm, and the lateral distance was 0.5 (n = 24), 5.0 (n = 13), and 10.0 cm (n = 12) for each group. The 13 ICD with live monitoring were all at a 0.5 cm lateral distance

*ICD* Implantable cardioverter defibrillator, *PBT* proton beam therapy, *PM* pacemaker

**Table 4:** Outcomes of in the vivo studies included

Study	Outcomes								
Authors	Field-to-generator distance, range (cm)	Distance between CIED and PTV (median and range)	CIED programming	Atrial lead	LV lead	CIED reprogramming before RT	Malfunctions	Clinical symptoms	Intervention
Oshiro et al. [29]	6–30 cm	NA	6 VVI, 1 VDD, 1 DDD	1	0	NA	3 resets in 2 patients, 2 detected through ECG	None	1 reprogramming
Gomez et al. [30]	0.8–40 cm	10 cm (range 0.8–40 cm)	NA	NA	NA	Yes, to detect a reset from the pulse rate	5 CIED reset in 4 patients, 1 ERI condition	None	5 reprogramming, 1 generator replacement
Ueyama et al. [31]	13 cm-> 50 cm	NA	6 DDD, 1 AAI-DDD, 2 rate response on	7	0	NA	2 CIED resets detected only through CIED check (2/7, 29%)	None	1 reprogramming
Seidens et al [32]	Distance between beam and CIED on BEV: 0 cm and 0.5 cm	13.4 cm (range 4.1–17.9 cm)	NA	NA	NA	None	1 enhanced lead impedance (fluctuating), (1/31, 3%)	None	None
Hashimoto T et al. [33]	Provided only for patients with thoracic (lung) tumors: 0–25 cm	NA	NA	NA	1/69	NA	7 resets in 5 patients, 3 oversensing in 1 patient	None	None
Overall population, *n* = 157	Range 0 to > 50 cm	Available range 0.8–40 cm	Mostly NA	Mostly NA	1/84 (1%)	Mostly NA	22 malfunctions in 16 pts (incidence 10% of pts) 17 resets in 13 pts (8%) 3 oversensing in 1 pt (1%), one ERI condition (1%) and one enhanced lead impedance (1%)	None	7 reprogramming, 1 generator replacement

*AAI-DDD* Pacing system with switching capability from atrial demand pacing to dual chamber demand pacing;* ERI* elective replacement indicator;* PTV* planning target volume;* CIED* cardiac implantable electronic devices;* DDD* dual chamber demand pacing;* ICD* implantable cardioverter–defibrillator;* LV* left ventricular;* PM* pacemaker;* NA* not available in the original study;* VDD* dual chamber sensing with ventricular demand pacing; *VVI * ventricular demand pacing

### Population

#### In vivo studies

Five retrospective [29–33] cohort studies published between 2008 and 2021 were included. A total of 157 patients (age range between 43 and 97 years old) who underwent 161 courses or particle-beam RT were included in the studies. Overall, 53 patients (34%) received thoracic RT for a thoracic tumor, 73 patients (46%) suffered abdominal or pelvis tumor, 27 patients (17%) had head-and-neck and skull base tumors, and 4 patients (3%) suffered bone and soft tissue sarcoma.

Finally, 135 patients (86%) had a PM and 22 (14%) an ICD. 15/81 (18.5%) patients with this information available were pacing-dependent. Except for one study that did not provide these data [33], dose constraints to the CIED’s power generator were maintained within the manufacturer and guidelines recommended limits by all the other studies (maximum dose of 2 Gy (RBE) or less) and the CIED was never located within the treatment field. Hashimoto et al. [33] did not provide information concerning CIED checking and intrasession monitoring; in contrast, in the remaining studies, each CIED was checked in advance before the first session. The two largest studies with available data (*n* = 73 patients, 83%) adopted intrasession monitoring with an in-room video system [30, 32]; in the remaining two studies with this information available, a continuous electrocardiography (ECG) monitoring was applied during each treatment fraction. In two studies [31, 32] (total 38 patients, 43%), the CIED was checked after each session, in one study weekly (*n* = 8, 9%)[29], in the last study (*n* = 42, 48%) [30] at the beginning and the end of RT.

#### In vitro studies

Three experimental studies were included [34–36] whose characteristics are reported below:

1. Hashimoto et al. [34] reported an experimental set-up study that simulated PBT delivered by passive scattering technique to ICD carriers. Four ICDs were placed in the phantom (0.3 cm laterally and 3 cm distally) outside the RT field of 10 × 10 cm^2^ with a total dose in-field of 107 Gy over 10 sessions of irradiation.
2. Wootton et al. [35] investigated the proton dose perturbation due to a high-voltage coil on leads from ICDs in a water phantom reporting effects up to 20–35% as predicted by the treatment planning system (TPS) or measured using radiographic films.
3. Bjerre et al. [36] evaluated the risk of malfunctions of pencil beam PBT in 49 CIEDs (50% PMs and 50% ICDs) located at a different distance from the Bragg’s Peak and the risk of noise, pace inhibition, and inappropriate shock therapy in 13 devices (9 ICDs, 69%) with connected leads that were monitored live during consecutive irradiations.

### Intervention

#### In vivo studies

Most patients underwent PBT (124 patients, 79%), the remaining CIRT (32 patients, 20%), and one patient (1%) both. The total delivered dose ranged between 10 Gy(RBE) and 88 Gy(RBE).

#### In vitro studies

All studies [34–36] were performed with PBT.

### Comparison

No randomized clinical trials (RCT) are available.

### Outcomes

#### In vivo studies

None of the studies assessed the actual dose absorbed by the CIED. The distance between the RT field and the CIED generator ranged between 0 and > 50 cm. Two studies provide the distances between the CIED and the Planning Target Volume (PTV), in the range between 0.8 cm and 40 cm [30, 32]. In one study only, RT was preceded by phantom simulation that used the same CIED as those of the patients involved; that kind of simulation was able to predict 50% of reset cases [1 over 2]. The predictive value and the details of phantom stimulations are not reported for the 5 patients who did not develop CIED malfunctions in the same study. Only two studies reported the estimated maximum dose to the CIED of both protons and neutrons [30, 34]. Gomez et al. [30] calculated the neutron dose equivalent as a function of proton energy, aperture distance, field size, and width of the spread-out Bragg peak. Among the 42 patients of the study, the median estimated maximum proton and neutron doses to the CIED in all patients were 0.80 Gy(RBE) [range 0.13–2.1 Gy(RBE)] and 3.46 Sv (range 0.11–11 Sv). Among the three studies including ICD patients, only one (*n* = 31) [32] reported details about ICD programming management: asynchronous pacing stimulation or deactivation of anti-tachycardia treatments of ICD’s through reprogramming or magnet placement was not performed. Overall, 22 malfunctions were reported in 16/157 patients (overall incidence 10% of patients). The most frequently observed CIED malfunction was the reset to safety backup mode (17 cases in 13/157 patients, incidence 8% of patients), followed by 3 episodes of ICD oversensing (all in the same patient), one elective replacement indicator (ERI) and one enhanced impedance of the device lead which fluctuated. Notably, both the ERI status and the instability in the CIED lead impedance had already been detected before RT, with no additional unexpected changes occurring after RT. Therefore, the association with RT appears very unlikely. Only 2/10 of the reset cases with this information available were detected through the ECG, the remaining cases through CIED post-treatment check. All the 13 patients who had device resets were treated by passive scattering. None of the patients with CIED reset was pacing dependent; there was no need for urgent interventions, nor any patients developed symptoms. Overall, 7/10 cases (70%) of CIED reset with this information available were managed by CIED reprogramming; the remaining were transient and resolved either spontaneously (  = 1) [31] or after re-initialization of the CIED ( = 2) [29]. The patient with ERI status underwent uneventful elective generator replacement [30]; no further details were provided by Hashimoto et al. [33] concerning the patient who experienced 3 episodes of ICD oversensing, except for the fact that no permanent device malfunctions were observed.

Limited sub-analyses were performed according to the radiation site (thoracic vs not thoracic), the type of device (PM vs ICD), the type of beam scattering, the type of particle (passive scattering PBT vs CIRT) and the field to generation distance. For the purpose of this analysis, the cases of ERI and of lead impedance fluctuation, both unlikely to be related to RT because already existent before RT start, were not considered. The incidence of malfunctions (total *n* = 14 patients) was 0/41 (0%) among patients receiving active scanning beams as opposed to 14/116 (12%) among patients receiving passive scattering therapy (*p* for comparison = 0.02). Passive scattering PBT was not associated with a significantly higher rate of malfunctions as compared to passive scattering CIRT (14/104, 14% vs. 0/23, 0%, p for comparison = 0.07). No significant differences were found either according to the type of device: 10/135 (7%) in PM recipients versus 4/22 (18%) in ICD recipients (*p* for comparison = 0.111). Incidence of malfunction according to the site of RT was 8/53 (15%) for patients receiving thoracic RT as opposed to 6/104 (6%) for patients receiving not thoracic RT (p for comparison = 0.07). Punctual data concerning the field to generator distance within each patient were only available for 3 proton studies (*n* = 18 patients, all treated with passive scattering). The mean field to generator distance was 13.3 ± 11.6 cm in patients with CIED reset (*n* = 8) as opposed to 22.1 ± 14.3 in patients with no device malfunctions (p for comparison = 0.196). Hashimoto et al. [33] reported the distance between the edge of the irradiation field and the CIED only for the 20 patients with thoracic tumors: the incidence of malfunctions was 0/12 (0%) among patients with a distance between 0 and 15 cm, as compared to 2/8 (25%) among those with a distance > 15 cm (range 15–25, p for comparison = 0.147). In the study of Gomez et al. [30], among the 4 cases of reset, the mean maximum proton and neutron doses to the CIED generator were 0.745 Gy(RBE) and 655 mSv, respectively. Malfunctions happened at different cumulative delivered doses, ranging from 4 Gy(RBE) to 67.5 Gy(RBE) near the end of the PBT. Except for one study where this information was not available [33], all the other 10 cases of CIED resets took place at neutron doses to the device of at least 300 mSv.

#### In vitro studies

In the study of Hashimoto et al. [34], no permanent malfunctions were observed using passive scattering PBT. Overall, 29 soft errors occurred over 40 sessions (incidence 73% per session) with a rate of 1 soft error per 15 Gy and a rate of power-on resets (changes to safety back-up mode) of 1 per 50 Gy. The calculated dose of secondary scattered neutrons per 1 Gy was 1.3–8.9 mSv/Gy.

Wootton et al. [35] showed a significant potential for perturbation in the delivered PBT when the ICD high voltage lead was not moving, while movements miming the cardiorespiratory ones consistently reduced (albeit not eliminated) the potential for perturbation.

Finally, Bjerre et al. [36] described 61 reset errors (60 in Biotronik and 1 in Boston Scientific devices, none in Medtronic and St. Jude Medical devices) over 1728 fractions of active scanning PBT, with an overall incidence of reset of 4.9%, 2.8% and 1.6% per fraction at a lateral distance of 0.5, 5.0 and 10.0 cm, respectively. The risk was higher for each group for Biotronik device carriers (19.4%, 5.1%, and 3.2%, respectively). The risk was constant throughout the sessions, and, except for the 0.5 cm group, higher for ICDs than PMs. While all the resets that occurred in Biotronik devices were reprogrammed to normal function, the single error that occurred in the Boston Scientific device was permanent (the device was locked in permanent safety mode). Secondary neutron dose significantly augmented the odds of CIED resets by 55% per mSv. Battery depletion was observed in 5 devices (all Medtronic ICDs), albeit several conditions may have disrupted the calculations in these explanted evices. Finally, no cases of noise, over- or undersensing, pace inhibition or inappropriate shock therapy occurred during 362 fractions of live monitoring.

When comparing passive scattering PBT [33] with active scanning PBT [35] at a similar lateral distance (0.3 and 0.5 cm, respectively), the incidence of malfunctioning (all soft errors except for one) per session was significantly higher with passive scattering PBT (29/40, 73% vs. 42/864, 4.9%, *p* < 0.00001). The overall incidence of mulfunctionings with a lateral distance within 0.5 cm was 7.9% (71/904).

## Discussion

To the best of our knowledge, our review represents the first study specifically addressing the topic of CIEDs malfunction associated with PBT and CIRT and including both in vivo and in vitro data. Concerning the key questions analyzed in the current review, according to PICO criteria, it emerges that:

- Query 1: In vitro data are scant and only two studies [34, 36] were found using PBT and showing an important soft error potential with passing scattering PBT, and a significantly lower, but still not trivial, particularly for Biotronik devices, soft error potential with active scanning PBT. Notably, in the study using active scanning, no cases of intrasession noise, over- or undersensing, pace inhibition or inappropriate shock therapy occurred. A potential for battery depletion was reported for active scanning PBT, albeit this needs to be confirmed in vivo since several in vitro factors may have contributed. The last in vitro study [35] showed a potential for a mild perturbation of a moving high-voltage lead on the delivered proton beam.
- Query 2: a total of 22 episodes of CIED malfunctions were observed in 16/157 patients undergoing 161 RT courses (overall incidence 10% of patients, 9% of RT courses), including 20 cases of CIED reset in 13 patients (incidence 8% of patients), 3 episodes of ICD oversensing (all in the same patient), one ERI condition and one increased (albeit fluctuating) lead impedance. The last two malfunctions were both pre-existing conditions before the beginning of RT sessions, and they were excluded from the sub-analyses on potential factors associated with CIED malfunctions. Therefore, no PBT/CIRT-related permanent malfunctions were observed (only resets or transient oversensing).
- Query 3: no patients developed clinical symptoms during the reset episodes, which were mostly detected through ICD checks. Only one study provided information about the year of CIED implantation, while only the largest one [33] provided full details about the type of PM/ICDs for the patients with malfunctions and the presence/absence of a left ventricle lead.
- Query 4: none of the 41 patients treated with active scanning developed CIED malfunctions, as opposed to 12% of patients receiving passive scattering therapy. A trend toward a higher incidence of CIED malfunctions was also experienced in thoracic vs not thoracic particle therapy as well as in passive scattering PBT versus passive scattering CIRT. No differences were observed according to the type of device (PM versus ICD).

The analyzed studies feature several limitations. Except for the multicentric study of Hashimoto et al. [33], the remaining studies represent single-center experiences, often with limited sample size. Moreover, patients and treatments were heterogeneous, and most studies lack details concerning the year of CIED implantation, the type of PM/ICDs and the number of leads (single-chamber versus dual-chamber versus cardiac resynchronization devices with a left ventricle lead). The latter might potentially influence both the incidence of hard errors due to lead damages as well as the patient’s symptoms during reset.

In the clinical studies, no differences were observed according to the type of device (PM versus ICD), but these findings might have been influenced by the inclusion of the latest ICD types, which were shown to be less sensitive to secondary neutrons scattering than the older ones [37]. Yet, the in vitro study [36] suggests a higher risk of ICDs than PMs. Concerning the type of device, an extensive review [38] predominantly including RT studies with photons and electrons (with only two PBT studies) showed that CIED malfunctions occur approximately in 3% of RT courses (with electrical reset being the most common ones). In the largest clinical study included in the review (560 CIED patients, 73 with ICD, photon RT only), Zaremba et al. [3] report a trend toward an increased risk of malfunctions in ICDs (6.8%) compared with PMs (2.5%). Subsequently, Aslian et al. [39] reported 6.9% of CIED malfunctioning during stereotactic RT. These data are in line with previous clinical reports [40]. Notably, in the large study by Zaremba [38], the location of the tumor was a strong predictor of CIED malfunction at univariable analysis, but its effect consistently declined after adjustment for beam energy [38]. This result supports the concept that soft errors such as electrical resets are mostly related to beam energy and secondary neutron scattering independently from the direct exposure to ionizing radiation. Secondary neutrons have been previously measured by various detectors [19] or estimated by Monte Carlo simulation [20, 41] using phantoms that are designed to represent human tissue. As already mentioned, the rate of neutron production in particle RT strongly depends on the dose delivery modality, and passive scattering yields much higher rates of out-of-field scattered neutrons, whereas active scanning allows for a significant dose advantage in neutron productions. This advantage has been estimated as a factor of at least 10 [42] and potentially even more (between 30 and 45) [43]. An Italian group recently reported the first in man case of active scanning PBT (single dose of 25 Gy-RBE), for the treatment of refractory ventricular tachycardia in a non-oncological PM-dependent patient with a biventricular ICD. No malfunctions were observed despite intensive intrafraction and post-therapy monitoring [44]. Yet, it has to be acknowledged that, as opposed to the very good safety profile emerged so far from the limited clinical experience of active scanning PBT [30, 32], pre-clinical studies mandate caution for the risk of reset (particularly for Biotronik devices) and the potential for battery depletion, while being extremely reassuring on the zero risk of more severe types of intrafraction malfunctionings such as pace inhibition or inappropriate shock therapy. Notably, there were only 4 patients with Biotronik devices in the clinical study by Seidensaal et al. [32].

PBT, based on the expected higher number of secondary scattered neutrons produced by protons as compared to photons with energies below 10 MeV, is considered at intermediate/high risk of CIED malfunction by all the consensus documents written so far [13–15, 17], while the management of patients receiving RT with CIRT is not specifically addressed. When comparing carbon ions with protons, several factors must be considered. On one side, neutron rate production with CIRT is expected to be higher due to nuclear fragmentation [45]. On the other side, the lower scattering both in air and in tissues of carbon ions, compared to protons, should lead to a reduction in the low dose region surrounding the target where presumably the device can be found. For these types of ion species, same for helium or oxygen, for instance, further investigations both in terms of real patient treatments and Monte Carlo simulation are needed.

### Practical implications and discrepancies among consensus documents

From a practical standpoint, patients considered at high risk of RT-induced CIED malfunctions require a closer level of monitoring. The 2017 HRS expert consensus statement [14] recommends performing a continuous visual and voice contact during each RT fraction in all CIED patients receiving RT, but a strong recommendation for a weekly complete CIED evaluation was made only for patients undergoing neutron-producing treatment. For the other patients, a complete CIED evaluation was recommended after the end of the course of RT. No specific recommendations were provided concerning the eventual need for and the timing of ECG monitoring or intrasession pulse monitoring. On the other side, according to the 2019 AAPM TG-203 report [15], CIED patients receiving neutron-producing RT should undergo ECG weekly monitoring, but there are no specific recommendations concerning intersession CIED monitoring. Finally, an Italian consensus document [13] recommends, for patients receiving PBT, intrasession ECG or pulse oximeter plus audiovisual monitoring, CIED in office or remote evaluation after the first session and then weekly. The same approach is shared by the recent European consensus document [16].

Based on the clinical studies included in the present review, 20% of the reset cases were detected through the ECG, the remaining cases through CIED post-treatment check. Notably, in the only study [29] where two reset cases were detected through the ECG, the CIEDs were checked weekly. On the contrary, in the other three studies with this information available [30–32] including the one published in 2019 [32] CIEDs were checked after each session; moreover, in the study by Gomez et al. [30], CIEDs were also re-programmed in advance (before each session) to detect a reset from the pulse rate.

Due to the high incidence of reset to safety backup mode in patients receiving passive scattering heavy-ions RT based on studies with intensive ICD checking, it would seem reasonable to implement the same kind of monitoring, including at least a CIED check after each session in all patients. A continuous intrasession ECG or pulse monitoring (in addition to the visual and voice contact) could be limited to pacing-dependent and ICD patients, particularly in case of previous ventricular arrhythmias. The management of patients receiving last generation, active scanning heavy-ions RT (both PBT and CIRT) is more controversial, due to the actual discrepancy between the limited clinical data, suggesting an almost zero risk of malfunctions, and in vitro data (based on PBT only) showing an overall low but not null potential for reset events, almost exclusively in Biotronik device carriers, and for battery depletion. Based on in vitro data, patients with a Biotronik devices, those with an ICD and those pacing dependent should probably be checked after each session, while the other patients could receive weekly complete in person CIED evaluations combined to remote CIED monitoring to collect safety data before and after each session.

## Conclusions

The available clinical and preclinical data consistently suggest a significant potential for neutron-related, not severe, electrical CIED malfunctions (asymptomatic resets) in patients receiving passive scattering particle RT. Accordingly, all these patients should be managed as a high-risk category until additional predictors or modulators of CIED malfunction risk are identified. On the contrary, clinical data on patients receiving the last generation, active scanning particle RT, albeit limited, disclose a promising safety profile, with no cases of CIED malfunctions reported so far. Yet, in vitro data still mandate caution for the risk of reset and potentially of battery depletion related to active scanning particle RT. Overall, the management of CIED patients receiving particle therapy is still extremely heterogeneous across centers. The safety profile of particle RT for CIED carriers may change in the next future due to technological improvements in both CIEDs and particle therapy, that is seeing the ongoing construction of several new centers worldwide. Still, it emerges an urgent call for shared protocols, as well as minimum criteria for scientific reporting.

## Supplementary Information

Below is the link to the electronic supplementary material.

Supplementary file1 (DOCX 18 KB)
