# Supplementary material for: The impact of particle radiotherapy on the functioning of cardiac implantable electronic devices: a systematic review of in vitro and in vivo studies according to PICO criteria
Source: Radiol Med. 2022 Jul 24;127(9):1046–58. doi: 10.1007/s11547-022-01520-6 (PMC9508006; doi:10.1007/s11547-022-01520-6)
Supplement: Supplementary file 1 — Supplementary file1 (DOCX 18 KB) [file 11547_2022_1520_MOESM1_ESM.docx]

**Supplemental Table 1 Risk of bias in included studies**

| Study | Bias across studies | | | | | | Source of funding and conflict of interest of authors of included studies | | |
| --- | --- | --- | --- | --- | --- | --- | --- | --- | --- |
|  | Bias arising from the process | Bias due to deviations from intended interventions | Bas due to missing outcome data | Bias in measurement of the outcome | Bias in selection of the reported result. | Overall risk | Trial design | Trial’s effect estimate | Synthesis of trial results |
| Oshiro et al., 2008 (29) | Low risk of bias | Some concerns | Low risk of bias | Low risk of bias | Low risk of bias | Low risk of bias | Low risk of bias | Low risk of bias | Low risk of bias |
| Gomez et al., 2013 (30) | Low risk of bias | Some concerns | Low risk of bias | Low risk of bias | Low risk of bias | Low risk of bias | Low risk of bias | Low risk of bias | Low risk of bias |
| Ueyama et al., 2016 (31) | Low risk of bias | Some concerns | Low risk of bias | Some concerns | Some concerns | Low risk of bias | Low risk of bias | Low risk of bias | Low risk of bias |
| Seidensaal et al., 2019 (32) | Low risk of bias | Some concerns | Low risk of bias | Low risk of bias | Low risk of bias | Low risk of bias | Low risk of bias | Low risk of bias | Low risk of bias |
| Hashimoto et al., 2021(33) | Low risk of bias | Some concerns | Some concerns | Some concerns | Some concerns | Low risk of bias | Low risk of bias | Low risk of bias | Low risk of bias |
| Hashimoto et al., 2012 (34) | Low risk of bias | Low risk of bias | Low risk of bias | Some concerns | Low risk of bias | Low risk of bias | Low risk of bias | Low risk of bias | Low risk of bias |
| Wootton et al., 2012 (35) | Low risk of bias | Low risk of bias | Low risk of bias | Some concerns | Some concerns | Low risk of bias | Low risk of bias | Low risk of bias | Low risk of bias |
| Bjerre et al 2021 (36) | Low risk of bias | Low risk of bias | Low risk of bias | Low risk of bias | Low risk of bias | Low risk of bias | Low risk of bias | Low risk of bias | Low risk of bias |
